# Pluripotent stem cells as a model to study non-coding RNAs function in human neurogenesis

**DOI:** 10.3389/fncel.2013.00140

**Published:** 2013-08-27

**Authors:** Alexandra Benchoua, Marc Peschanski

**Affiliations:** ^1^Centre d’Etude des Cellules Souches, Institut des cellules Souches pour le Traitement et l’Étude des Maladies monogéniques, Association Française contre les MyopathiesEvry, France; ^2^INSERM/UEVE UMR 86, Institut des cellules Souches pour le Traitement et l’Étude des Maladies monogéniques, Association Française contre les MyopathiesEvry, France

**Keywords:** pluripotent stem cells, micro-RNA, neurogenesis, neuro-developmental diseases, psychiatry

## Abstract

As fine regulators of gene expression, non-coding RNAs, and more particularly micro-RNAs (miRNAs), have emerged as key players in the development of the nervous system. *In vivo* experiments manipulating miRNAs expression as neurogenesis proceeds are very challenging in the mammalian embryo and totally impossible in the human. Human pluripotent stem cells (hPSCs), from embryonic origin (hESCs) or induced from adult somatic cells (iPSCs), represent an opportunity to study the role of miRNAs in the earliest steps of human neurogenesis in both physiological and pathological contexts. Robust protocols are now available to convert pluripotent stem cells into several sub-types of fully functional neurons, recapitulating key developmental milestones along differentiation. This provides a convenient cellular system for dissecting the role of miRNAs in phenotypic transitions critical to brain development and plasticity that may be impaired in neurological diseases with onset during development. The aim of this review is to illustrate how hPSCs can be used to recapitulate early steps of human neurogenesis and summarize recent reports of their contribution to the study of the role of miRNA in regulating development of the nervous system.

## INTRODUCTION

Human neurogenesis is the result of a tightly controlled sequence of events that associates environmental signals and intra-cellular molecular mechanisms. Impaired neurogenesis is at the origin of the so-called neuro-developmental disorders, such as autism spectrum disorders (ASDs) or Down’s syndrome, and is thought to be involved in the etiology of psychiatric disorders such as schizophrenia or bipolar disorders (Moreno-De-Luca et al., 2013). Micro-RNAs (miRNAs) are abundant, short-lived, double strand non-coding RNAs (nc-RNAs) of 22 nucleotides that act as post-transcriptional repressors targeting multiple mRNAs (Yates et al., 2013). They account for an additional level of intricacy to gene regulation and, therefore, represent attractive candidates to interpret subtle developmental regulations. Genetic manipulation of the miRNAs machinery in rodent models severely impaired several aspect of neurogenesis (Sun et al., 2013). However, to evaluate whether this may be relevant to human neurogenesis, both physiologically and in a pathological context, these experiments need to be replicated and supplemented in human models of neurogenesis.

Over the last decade, human pluripotent stem cells (hPSCs) have emerged as powerful tools with the potential to further illuminate key mechanisms underlying neuronal development (Chamberlain et al., 2008; Crook and Kobayashi, 2008). These cells allow investigators to more specifically assess aspects of human neurogenesis that were previously hardly attainable due to technical obstacles at accessing human embryonic and fetal tissues. Pluripotent stem cell (PSC) can be obtained from the embryo inner cell mass or reprogramed from any adult somatic cells (Gokhale and Andrews, 2012). Their self-renewal property offers the opportunity to amplify them until reaching the cell mass necessary to perform large throughput studies including miRNA whole genome profiling. As PSCs, they can virtually give rise *in vitro* to any cell type of the human body including neurons. Of interest, the differentiation paradigms of hPSC into different sub-types of neurons recapitulates the key milestones of human neurogenesis including: (i) early neural commitment and neuro-epithelial cells differentiation, (ii) regionalization of the early neuro-epithelial cells into more specialized neural progenitors, (iii) terminal differentiation of the specialized progenitors into specific sub-types of neurons and maturation of these neurons until the formation of functional synapses and complex networks. The involvement of miRNAs in each milestone can therefore be assessed functionally using genetic manipulation to achieve loss or gain of function into the cells.

While human embryonic stem cells (hESCs) remain the gold standard to study the physiological aspects of early human development, PSCs reprogramed from adult somatic cells (iPSC) offer the opportunity to address important issues regarding miRNA participation to disease etiology in a patient-related genetic background. Here, we summarize the different steps of human neurogenesis that can be recapitulated from hPSC and the current protocols that have been implemented to obtain them. We will next review the current knowledge regarding miRNA-dependent regulation in PSC-derived models of neurogenesis. Finally, we will discuss the current success of iPSC derivation from patient with neuro-developmental or early onset psychiatric disorders and how these new cellular models could help increasing our understanding about the involvement of miRNAs in human diseases.

## PLURIPOTENT STEM CELLS AS A CELLULAR SYSTEM TO RECAPITULATE KEY STEPS OF EARLY HUMAN NEUROGENESIS

### ENGAGEMENT OF PLURIPOTENT STEM CELLS INTO THE NEURAL LINEAGE

The earlier event of human neurogenesis that can be modeled *in vitro* using PSC is the conversion of PSCs into the neural lineage to form the first population of neural progenitors found in the neural plate, the neuro-epithelial cells. hPSC may be coaxed along the neural lineage and differentiate into a population of bipolar neuro-epithelial cells that express the main markers of the neural tube, Sox1 and Sox2, and organize in rosette-like multicellular structures using different methods. One efficient and convenient protocol was revealed in parallel by Chambers et al. (2009) and our group (Boissart et al., 2012). PSC are first cultivated as a monolayer then the medium changed to a neural induction medium containing a combination of inhibitors of both the bone morphogenetic proteins (BMPs) and transforming growth factor-beta (TGF-β), Smad-dependent, pathways. After 10–15 days, the neural conversion of PSC into Pax6/Sox1 positive neuro-epithelial cells is fully achieved. When used to inhibit TGF-β-mediated pluripotency networks, the small molecule SB431542 promotes exit of cells from the pluripotent compartment and suppresses mesendodermal fates by inhibiting endogenous activin and nodal signals. Neural conversion of the resulting ectodermal cells was achieved with addition of the BMP inhibitor Noggin (Chambers et al., 2009; Boissart et al., 2012). The resulting neuro-epithelial cells are competent to form neural “rosettes” that morphologically mimic the neural tube cells and could further be differentiated into different sub-types of functional neurons. Clear advantage of this system is its dramatic efficiency and relative simplicity. The efficiency of the phenotypic transition between pluripotency and neural commitment can be easily monitored in real-time following the morphological changes characteristic of the formation of the neuro-epithelium, the so-called neural “rosettes.” Quantification can be achieved measuring the number of cells expressing the canonical neural markers Sox1 and Pax6 but down-regulating the pluripotency markers Oct-4 and Nanog. This allows the differential large-scale profiling of miRNA expression since both pluripotent and neuro-epithelial cells can be obtain to near-purity. Finally, the monolayer culture mode is perfectly adapted to transfection methods and functional validation can easily be conducted (Boissart et al., 2012). Neural commitment can also be achieved using embryoid bodies (EBs) where PSCs are differentiated *in vitro* by spontaneously self-assembling in suspension into 3D cell aggregates. This technique of differentiation promotes the formation of the three embryonic germ layers in parallel. It can be more challenging to manipulate these 3D structures than cells differentiated as monolayer; however, the contribution of a given miRNA to influence the balance between the different embryonic fates can be addressed (Xu et al., 2009).

### REGIONAL PATTERNING OF NEURO-EPITHELIAL CELLS

In addition to the acquisition of an early neural fate, neuro-epithelial cells will progressively adopt a specific regional identity along the neural tube axes in response to exogenous factors. The resulting cells have a more restricted potential and produce only specific sub-types of neurons according to their position along the rostro-caudal and dorso-ventral (DV) axis. Fundamental to the existence of divergent structures in the brain is the early region-specific molecular programing. In mammal embryos, the anterio-posterior (AP) axis is specified as neural commitment proceeds. The closing neural tube quickly divides into three primary vesicles: the anterior forebrain, the midbrain, and the posterior hindbrain. The forebrain will further sub-divide into two structures, the rostral telencephalon and the diencephalon (Pombero and Martinez, 2009), whereas the caudal hindbrain will form the rhombencephalon and the spinal cord. Secondary patterning sequences will further specify DV domains inside each structure (Lupo et al., 2006). The organization of these secondary vesicles prefigures the future brain structures. The telencephalon will give rise to the cortex in its dorsal part and to basal ganglia in its ventral part. The thalamus and hypothalamus will emerge from the ventral diencephalon, the substantia nigra from the ventral mesencephalon, the cerebellum from the rhombencephalon, spinal motor neurons will form from the ventral part of the spinal cord whereas sensorial neurons will differentiate from the dorsal part (de Graaf-Peters and Hadders-Algra, 2006).

*In vitro* regionalization of PSC-derived neuro-epithelial cells has been achieved successfully for some representative neuronal populations by translating knowledge from embryogenesis. During embryogenesis, regional patterning is under the control of extra-cellular signals that provide a group of neural progenitors with the unique competency to produce specific neuronal sub-types. The activity of these signals is spatiotemporally integrated by neural progenitors to determine the specific combinations of transcription factors activated in distinct AP and DV compartments of the central nervous system (CNS; Vieira et al., 2010). Master factors include Wnt, sonic hedgehog (SHH), fibroblast growth factors (FGFs), BMPs, and retinoic acid (RA) acting in gradient of concentrations. Anterior fates have been obtained from PSC using default protocols, in the absence of any morphogen (Li et al., 2009; Zeng et al., 2010). However, the efficiency can be greatly improved by using inhibitors of the Wnt pathway. The resulting population of neural progenitors expresses high levels of the anterior marker FoxG1 and can be further patterned dorsally or ventrally using the interplay between Wnt and SHH pathways. Further differentiation of primitive neuro-epithelial cells in the absence of SHH spontaneously produce neural progenitors that express the dorsal markers Pax6 and Emx1 and will ultimately give rise to glutamatergic projection neurons of the different cortical layers (Shi et al., 2012). In contrast, gradual activation of SHH-dependent pathway allows the successful production of progenitors from the ventral ganglionic eminence, expressing the markers Nkx2.1 or Gsh-2, and the corresponding cortical or striatal GABAergic interneurons (Carri et al., 2013; Maroof et al., 2013).

Interplays between RA, FGF-8, and Wnt pathways promote more caudal fates. Midbrain dopaminergic (mDA) neurons of the substantia nigra and the ventral tegmental area emerge from progenitors located in the floor plate of the ventral part of the embryonic midbrain (Ono et al., 2007). Accordingly, most protocols that aim at deriving DA neurons progenitors from hPSC rely on the exposition of early neuro-epithelial to SHH and to FGF-8, a weak but sufficient caudalizing factor (Perrier et al., 2004; Andersson et al., 2006; Friling et al., 2009; Rhee et al., 2011). Recently, the small molecule CHIR99021, a glycogen synthase kinase-3 inhibitor that mimics Wnt pathway activation, has been identified as a more potent caudalizing agent than FGF-8, and has been used to produce high yields of mDA neurons in combination with a modified form of SHH (Kirkeby et al., 2011; Kriks et al., 2011). Spinal cord motor neurons originate from the motoneuron progenitor domain located in the ventral developing spinal cord (Soula et al., 2001). In order to obtain efficient spinal motor neurons *in vitro*, primitive neuro-epithelial cells need to be caudalized as the neural induction proceeds, using high concentrations of RA then ventralized using SHH (Chipman et al., 2012; Takazawa et al., 2012).

**FIGURE 1:**
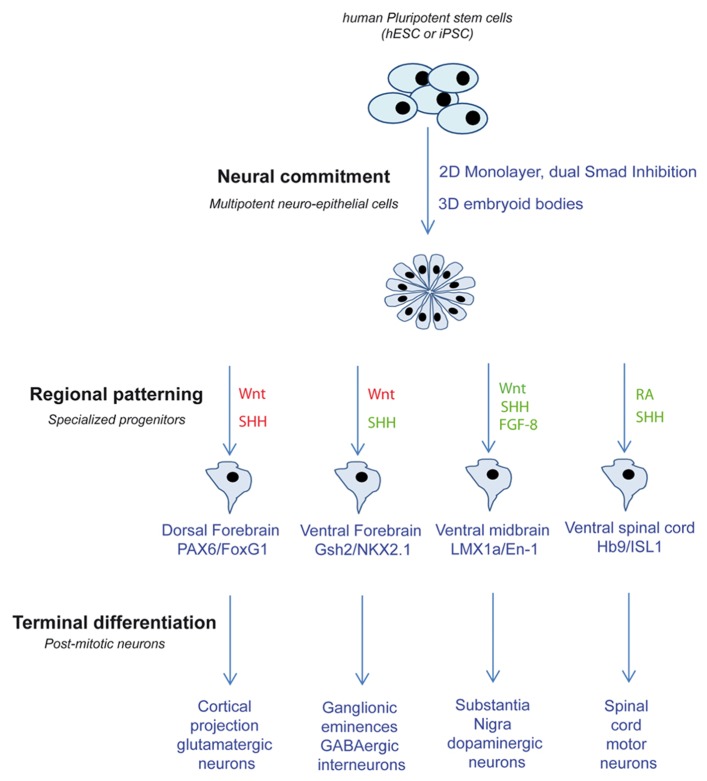
**Schematic depicting the different strategies used to produce different neuronal sub-types from PSC.** Related pathway activations are in green and inhibitions are in red. SHH, sonic hedgehog; FGF-8, fibroblast growth factors 8; RA, retinoic acid.

Most of these protocols of directed differentiation are efficient enough to yield largely enriched population of a given progenitor sub-type (**Figure 1**). Differential miRNA profiling experiments comparing different progenitor populations differentiated from the same neuro-epithelial cells can help identifying miRNAs specifically involved in progenitor specification. In addition, the involvement of miRNA in progenitor response to patterning molecules can easily be assessed in a dose-dependent manner.

### *IN VITRO* MODELING OF THE BALANCE BETWEEN SELF-RENEWAL AND NEURONAL DIFFERENTIATION

During embryogenesis, progenitors committed to the neural lineage and regionalized undergo several rounds of symmetric divisions (self-renewal) before giving rise to terminally differentiated post-mitotic neurons. This active phase of self-renewal is necessary to constitute then maintain a large pool of progenitors. In addition, for many brain structures, waves of terminal differentiation occur at different time points allowing the genesis of different sub-types of neurons from the same starting progenitors (McConnell, 1995). This is particularly illustrated by the formation of the mammal neocortex layers. Glutamatergic projection neurons of the neocortex organize as six layers in the human brain. All layers are formed from the same pool of progenitors located in the cortical sub-ventricular zone. However, early born neurons will form the deeper layers (V–VI) whereas later born neurons will constitute the upper layers (II–IV; Rakic, 1974; Frantz and McConnell, 1996). Studying the regulation of this balance between self-renewal and terminal differentiation is therefore particularly meaningful to improve our ability to grow PSC-derived neural progenitors *in vitro* in order to control the production of relevant neuronal sub-types but also because subtle disturbance of this balance may be involved in the genesis of many neuro-developmental diseases.

Pluripotent stem cell-derived self-renewing neural progenitors can be obtained by placing the early neural rosettes, which contain neuro-epithelial cells, in culture media supplemented with mitogenic factors. When cultivated in presence of Notch ligands and SHH, early neuro-epithelial cells retain both the morphological organization (epithelial structures) and molecular signature of naïve neuro-epithelial cells (Elkabetz et al., 2008). These rosette-derived neural stem cells (R-NSC) can be maintained upon several rounds of symmetric division and produce different sub-types of neurons after a short exposure to relevant patterning molecules. Successful amplification of neuro-epithelial was also achieved using FGF-2 alone or in combination with epidermal growth factor (Delaloy et al., 2010; Falk et al., 2012) with cells responding at least in part to patterning molecules. It remains to establish whether the molecules used to amplify neural progenitors by the mean of self-renewal are relevant to the physiological situation. However, upon mitogens withdrawal, these neural progenitors quickly exit the proliferative compartment to engage the final program of differentiation as post-mitotic neurons. It is therefore possible to screen for miRNAs involved in the maintenance of the proliferative state or, in contrast, in the decision of terminal differentiation.

### SYNAPTOGENESIS AND FUNCTIONAL NETWORK

To be considered as fully functional, neurons have to form electrically active synapses and organize as complex neuronal networks. Formation of electrically functional synapses has been recorded in most of PSC-derived neuronal sub-types. Mixed population of forebrain neurons (which include GABAergic and glutamatergic neurons) can self-organize as a network forming functional synapses (Kim et al., 2011b). When the differentiation is directed to form cortical pyramidal neurons, formation of glutamatergic synapses can be monitored by measuring the assembly of cellular contact where presynaptic proteins are co-localized with PSD-95, a protein of the post-synaptic densities specific of glutamatergic synapses. Electrophysiology experiments showed neuronal responses to glutamate challenges. The recording of spontaneous action potentials indicated that these pyramidal neurons organized as complex autonomous networks (Shi et al., 2012). Telencephalic GABAergic interneurons, with a striatal or a cortical identity, also form spontaneously vesicular GABA transporter (VGAT)/gephyrin-expressing GABAergic synapses that are electrically active (Carri et al., 2013; Maroof et al., 2013). PSC-derived mesencephalic neurons efficiently release dopamine and exhibit spontaneous, network-mediated, electrical activity (Kriks et al., 2011). Finally, several studies reported that PSC-derived spinal cord motor neurons are able to form functional neuromuscular junctions when co-cultivated with myotubes (Umbach et al., 2012). In all these culture systems, synapse formation and activity can easily be monitored both morphologically, following clustering and co-expression of specific molecular markers, and functionally, measuring calcium entry in response to pharmacological stimulations or by the mean of electrophysiological recordings. This opens the path to functional studies of miRNA involvement in the very early steps of synaptogenesis as well as in modulating synaptic activity.

## PLURIPOTENT STEM CELLS TO STUDY miRNA FUNCTION IN PHYSIOLOGICAL NEUROGENESIS

**FIGURE 2:**
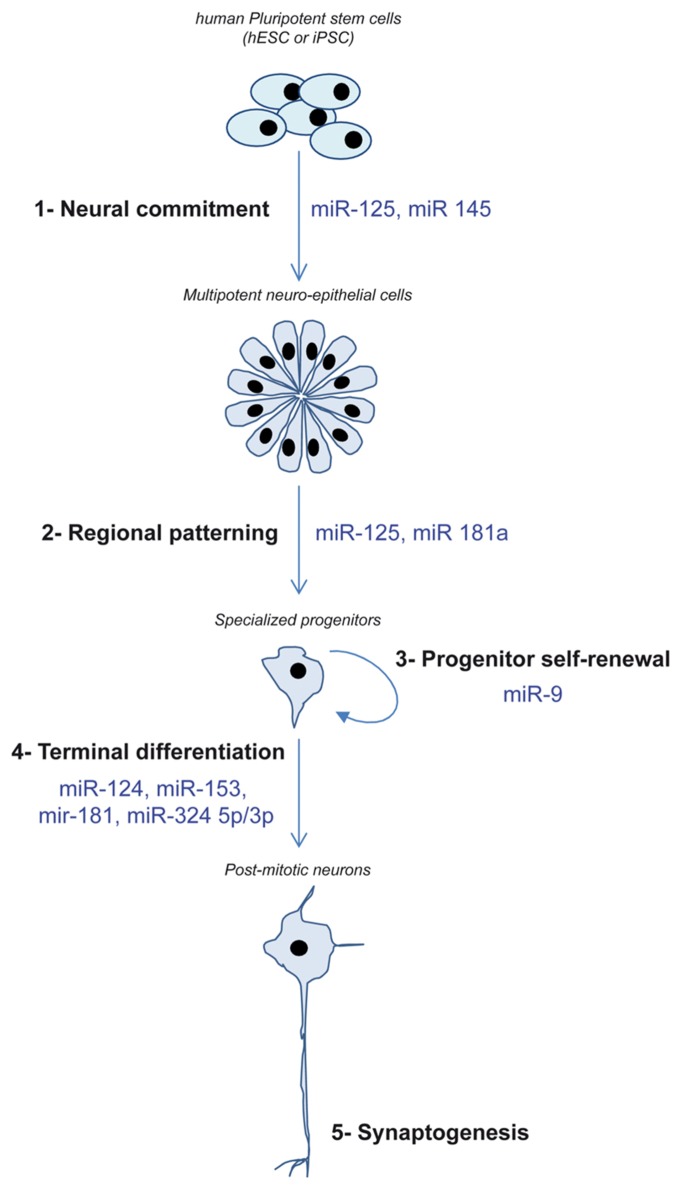
**Schematic depicting the different steps of neurogenesis recapitulated *in vitro* using hPSC and the miRNA demonstrated as regulators of these steps**.

So far, hPSC and their neural progeny have contributed to increase our knowledge regarding the involvement of miRNAs in two neurogenesis steps that are hardly accessible *in vivo*: the very early stage of engagement into the neural lineage and the governance of self-renewal/migration/neuronal differentiation of neural progenitors (**Figure 2**).

### miRNA REGULATING THE COMMITMENT INTO THE NEURAL

At least two miRNAs have been demonstrated to promote the conversion of PSCs into neuro-epithelial cells, miR-125 and miR-145. Xu et al. (2009) used the EBs model to identify miRNAs regulating hESC differentiation. Using a whole genome approach based on Taqman qPCR, they showed that miR-145 expression quickly increased as hESC enter the differentiation process. Forced expression of miR-145 favored the differentiation along the ectodermal lineage including the neural fate. In contrast, when miR-145 activity was blocked using locked nucleic acid (LNA)-antimiR oligos, endodermal differentiation occurred. The authors identified Oct-4 and KLF-4 as relevant targets of miR-145. miR-125 was also reported as a key regulator of hESC neural conversion. We induced the neural conversion of hESCs by treating them with the two Smad inhibitors: Noggin and SB431542 (Boissart et al., 2012). Using this model, the kinetics of activation of three brain-expressed miRNAs, miR-9, miR-124, and miR-125, was analyzed over time. Only miR-125 was found to be activated in a time window compatible with a role in the neural commitment decision. Functional studies confirmed that miR-125 activity was necessary to fully achieve an efficient engagement of hESC into the neural lineage by both promoting hESC differentiation and blocking alternative, non-neural fate choices. Silencing by miR-125 of Smad-4, the key co-factor of activin- and BMP-dependent Smad pathways, was central to its role in the promotion on neural commitment.

Next to miRNAs actively promoting neural conversion are miRNAs that block this critical decision in order to maintain the cells in a pluripotent state or foster alternative fates. miRNAs of the miR-302/miR-367 family are particularly enriched in PSC. miR-302/miR-367 target several endogenous inhibitors of BMP and activin pathways including Lefty, TOB2, DAZAP2, and SLAIN1. Consequently, Smad activity is elevated in cells expressing these miRNAs which render neural conversion impossible (Lipchina et al., 2011). More recently, miR-302 was shown to directly target NR2F2, a transcription factor involved in the very early triggering of the neural genetic program, suggesting that miR-302 may more specifically avoid a spontaneous commitment of PSCs into the neural lineage (Rosa and Brivanlou, 2011). Next to miR-302 family members, the miR-371/miR-372/miR-373 cluster is also considered as a potent inhibitor of the neural lineage commitment (Kim et al., 2011a). Expression of miR-371 is induced by the pluripotency-associated transcription factor KLF-4. PSC lines exhibiting high levels of endogenous miR-371 showed an altered neurogenic potential. Efficient neural conversion was restored upon miR-371 inhibition using LNA-antagomiR oligos demonstrating a causal role of miR-371 in repressing the neural fate. Interestingly, miR-371 activity did not compromise differentiation into other lineages but seemed rather specific of the neural lineage as it controlled the sensitivity of the PSC response to BMPs signal.

Taken together, the identification of miRNAs regulating the efficiency of PSCs neural conversion has highlighted the importance of the fine tuning of Smad-dependent pathways. miR-125, miR-302, and miR-371 both target proteins involved directly in signaling mediated by receptors of the TGF-beta family and modulate finely the strength of the signal transduction. miRNAs promoting the neural conversion target directly the Smad proteins whereas miRNAs favoring alternative fates contribute to secure the activation of these pathways by targeting their endogenous inhibitors. The hPSC model of neural differentiation has therefore contributed to illustrate how subtle the decision of lineage commitment is regulated.

### miRNA REGULATING PROLIFERATION AND DIFFERENTIATION OF NEURAL PROGENITORS

A crucial role of miRNAs in regulating the pool of neural progenitors has been well established in rodent models (Volvert et al., 2012). The brain-enriched miR-9 is mainly expressed in neurogenic areas during development suggesting its involvement in molecular mechanisms regulating self-renewal and proliferation of neural progenitors (Deo et al., 2006). Accordingly, miR-9 has been found highly expressed in self-renewing progenitors stably established from the multipotent and immature hESC-derived neuro-epithelial cells (Delaloy et al., 2010; Boissart et al., 2012). In hESC-derived progenitors amplified as neurospheres using FGF-2, miR-9 plays a crucial role in the maintenance of the capacity of proliferation and migration of these progenitors (Delaloy et al., 2010). Interestingly, the authors also assessed the role of miR-9 in the transition between immature multipotent neuro-epithelial cells and the fate restricted mature neural progenitors. They showed that miR-9 activity was essential to this maturation step by directly targeting the cytosolic protein Stathmin.

In contrast to the proliferation-promoting action of miR-9, several miRNAs have been identified as regulators of the decision of terminal neuronal differentiation. The neuronal-specific miR-124 was found enriched in culture of hPSC-derived post-mitotic neurons but not in the proliferative neural progenitors from which they were differentiated (Delaloy et al., 2010; Stappert et al., 2013). Functional studies showed that its forced expression increased the rate of neuronal differentiation whereas blocking its activity resulted in an impaired neuronal production (Stappert et al., 2013).

Next to the study of miRNAs already described as brain-specific, a differential, whole genome, miRNAs profiling was performed comparing self-renewing hESC-derived multipotent neuro-epithelial stem cells (lt-NES) to their neuronal progeny (Stappert et al., 2013). This extensive profiling pointed to additional miRNAs enriched in differentiated neurons, miR-125b, miR-153, miR-181a/181a*, and the cluster miR-324-5p/3p. Ectopic expression of miR-153, miR-181a/181a*, and miR-324-5p/3p shifted lt-NES cells from self-renewal to neuronal differentiation. lt-NES represent ventral hindbrain precursors that mainly give rise of GABAergic interneurons. However, they can also produce small amount of tyrosine hydroxylase (TH) neurons, an enzyme converting L-tyrosine into L-DOPA, considered a marker of catecholaminergic neurons. The authors asked whether, next to promoting neuronal differentiation, these miRNA can also influence the neuronal fate. miR-125b and miR-181a both increased the number of TH positive neurons. In contrast, miR-181a* inhibited the formation of TH neurons and promoted the production of GABAergic neuronal cells.

These pioneer studies illustrate how hPSC can greatly improve our knowledge about miRNAs involvement in physiological neurogenesis by helping elucidating the functional impact of miRNA activities and identifying their targets.

## DECIPHERING THE IMPACT OF miRNAs IN NEURO-DEVELOPMENTAL DISEASES USING PATIENT-DERIVED iPSCs

### THE iPSC BREAKTHROUGH

In 2007, Yamanaka and colleagues made the breakthrough discovery of a simple method to reprogram human somatic adult cells into fully PSCs, a type of cells now widely known as iPSCs (Takahashi et al., 2007). Since iPSC can be derived from virtually all nucleated cell types of the body, it suddenly removed the barriers raised until this date by the use of PSC derived from embryos that limited investigations to cells with unknown clinical status (the so-called wild-type cells) or to lines carrying mutations of the few diseases eligible for a pre-implantation genetic diagnosis (PGD). PSC induced from somatic cells offer the unlimited possibility to model neurogenesis from any patient including those for which the cause of the disease is not fully identified – e.g., multifactorial disorders – but with a well-documented clinical characterization (Bellin et al., 2012).

Although promising, the field of iPSC is still considered “at work” and questions remain regarding the accuracy of modeling disease with a strong epigenetic origin using genetically reprogramed cells. Indeed, concerns have been raised about the differences in genes expression, including miRNA profiles, between iPSC and hESC, suggesting that, next to the disease context, iPSC behavior may also be influenced by the technique and efficiency of reprograming as well as by the cell type from which the iPSC line was produced (Chin et al., 2009; Marchetto et al., 2009; Vitale et al., 2012). Consequently, iPSC may model neuro-developmental diseases with strong epigenetic components differently than hESC (Urbach et al., 2010). However, some evidences indicate that certain epigenetic marks are conserved with reprograming, including parental imprinting (Chamberlain et al., 2010; Yang et al., 2010). To date, several iPSC lines have been derived from patients with various neuro-developmental disorders including Rett’s syndrome (RS; Kim et al., 2011c), fragile X syndrome (FXS; Urbach et al., 2010), Down’s syndrome (Briggs et al., 2013; Weick et al., 2013), Timothy’s syndrome (Pasca et al., 2011), Angelman’s syndrome (Chamberlain et al., 2010), Prader–Willi’s syndrome (Yang et al., 2010), and Schizophrenia (Brennand et al., 2011). Some of those have been shown to recapitulate *in vitro* important features of the diseases which make them attractive tools to further study mechanisms leading to pathological phenotypes including the influence of miRNAs already described as dysregulated in brains of patients or in animal models (**Table 1**).

**Table 1:** Summary of iPSC lines in which the role of miRNAs dysregulated in animal models or human brains can be further investigated.

Disease	Origin	References of iPSC lines	Phenotype of iPSC-derived neurons	miRNAs of interest
Fragile X syndrome	Loss of function of FMRP (FMR1 gene)	Urbach et al. (2010), Sheridan et al. (2011)	Hyper-excitability of glutamatergic synapses	DICER and AGO-1 complexes
Rett’s syndrome	Loss of function of MeCP2 transcriptional repressor	Marchetto et al. (2010), Kim et al. (2011c), Cheung et al. (2012)	Decreased soma size, neurite atrophy, decreased efficiency of glutamatergic synapses	miR-132, miR-184, miR-483-5p, miR-212
Schizophrenia	Multifactorial	Urbach et al. (2010); Brennand et al. (2011), Paulsen Bda et al. (2012), Robicsek et al. (2013)	Diminished neuronal connectivity	miR-17-5p, miR-34a, miR-107, miR-122, miR-132, miR-134, miR-137
Down’s syndrome	Additional copy of chromosome 21	Briggs et al. (2013), Weick et al. (2013)	Reduced synaptic activity, increased sensitivity to oxidative stress	miR-99a, miR-125b, miR-155, miR-802, Ret 7c

### MONOGENIC SYNDROMES OF AUTISM SPECTRUM DISORDERS AND MENTAL RETARDATION

Micro-RNAs, as fine regulators of protein translation, influence directly the level of gene expression. A central role of synaptic gene dosage in the emergence of ASDs and mental retardation (MR) is now well established (Toro et al., 2010), suggesting that miRNAs studies in iPSC models may bring some light into the dark areas of these early onset neuro-developmental disorders. More particularly, understanding the link between genes responsible for monogenic forms of ASD/MR and the miRNA machinery may help understand why patients with the same genetic mutation in coding sequences can develop differentially severe symptoms. So far, iPSC have been successfully derived from individuals with two monogenic forms of ASD/MR: the RS and the FXS. RS is an inherited neuro-developmental disorder with an X-linked gene inheritance mainly affecting women. Most forms of RS are due to a loss of function mutation in the transcriptional repressor MeCP2 (methyl CpG binding protein 2; Ravn et al., 2011). RS iPSC replicate some prototypical features found in animal models including decreased neuronal soma size, neuritic atrophy and decreased efficiency of glutamatergic synapses (Marchetto et al., 2010; Kim et al., 2011c; Cheung et al., 2012). Disruption of MeCP2 gene in mice leads to the dysregulation of a set of miRNA potentially of influence in neurogenesis including miR-132, miR-184, miR-483-5p, and miR-212 (Nomura et al., 2008; Im et al., 2010; Urdinguio et al., 2010; Han et al., 2013). The role of each miRNA in the development of typical RS neuronal features can be addressed using iPSC-derived neurons. Similarly, several iPSC lines have been derived from individuals with the FXS. In FXS, abnormal expansion of a CGG triplet in the 5′UTR of the FMR1 gene leads to the defective translation of the FMR1 gene and to the loss of the resulting protein fragile X mental retardation protein (FMRP; Wijetunge et al., 2013). FXS iPSC lines-derived neurons recapitulate the typical hyper-excitability of glutamatergic synapses and developmental defects described in animal models (Urbach et al., 2010; Sheridan et al., 2011). FMRP has been shown to directly control miRNA biogenesis through direct interaction with DICER and AGO-1 complexes (Jin et al., 2004). Of interest, functional investigations of miRNAs regulated by MeCP2 or FMRP proteins in iPSC-derived neurogenesis models may help address whether the dysregulation of these miRNAs have a real impact on the strength of the pathologic phenotypes and whether this can be reversed.

### MULTIFACTORIAL SYNDROMES

One of the main interests of recapitulating neurogenesis with patient-derived iPSC is the opportunity to address the role of miRNAs in a human genetic background permissive to the development of multifactorial diseases such as schizophrenia or Down’s syndrome. Although psychiatric disorders, such as schizophrenia, affect several brain regions and produce a complex array of clinical symptoms, basic phenotypes likely exist at the level of single neurons and simple networks. Being highly heritable, it is hypothesized that these disorders are amenable to cell-based studies *in vitro* (Brennand et al., 2012). Accordingly, the human-induced PSC (hiPSC) technology makes it possible to study schizophrenia and other psychiatric disorders using live human neurons with a genetic predisposition without knowledge of the genes interacting to produce the disease state. Genome-wide profiling has listed a number of changes in miRNAs expression levels in the brain of patients with a diagnosis of schizophrenia (Kim et al., 2010; Moreau et al., 2011; Santarelli et al., 2011). These include miR-17-5p, miR-34a, miR-107, miR-122, the brain-specific miR-132, the synaptic miR-134, miR-185, miR-382, and miR-652. Interestingly, a single-nucleotide polymorphism in miR-137, a miRNA previously reported as a regulator of neuronal maturation, was consistently found to be one of the common alleles associated with a high risk of developing schizophrenia (Whalley et al., 2012). To date, several groups have obtained iPSC from individuals diagnosed with schizophrenia (Urbach et al., 2010; Brennand et al., 2011; Paulsen Bda et al., 2012; Robicsek et al., 2013) and have described impaired neurogenesis in these lines opening the path to further investigations regarding miRNA active participation to the initiation and the progression of the disease.

Another multifactorial disease for which hiPSC would be a valuable study tool is Down’s syndrome, also known as trisomy 21. In the human, Down’s syndrome sums MR, craniofacial morphological abnormalities and heart failure due to an additional copy of the long arm of chromosome 21 (Hsa21). Hsa21 contains approximately 552 genes, 166 of which are orthologous to genes localized in syntenic regions of three mouse chromosomes: Mmu16 (110 orthologous genes), Mmu17 (19 orthologous genes), and Mmu10 (37 orthologous genes; Rueda et al., 2012). Based on these homologies, several mouse models that are trisomic for different sets of Hsa21 genes have been developed but failed to properly recapitulate the complete neurological symptoms of Down’s syndrome, suggesting that Hsa21 might contain additional elements that are human-specific. Hsa21 has been predicted to contain at least five nc-RNAs, miR-99a, miR-125b, miR-155, miR-802, and Ret-7c (Kuhn et al., 2008). The recently published trisomic iPSC lines (Briggs et al., 2013; Weick et al., 2013) will probably help addressing the question of the importance of gene dosage, including miRNA, in the development of the neurological features of Down’s syndrome.

## CONCLUSION

By their ability to recapitulate human neurogenesis *in vitro* and their flexibility regarding genetic manipulation, hPSC have revealed valuable tools to help deciphering the role of miRNAs in the earlier events of human neurogenesis.

So far, hESC remain the “gold standard” to faithfully investigate the functional consequences of miRNA activity on different steps of human neurogenesis since they still represent the closest cellular model to the physiological situation. However, the iPSC have revolutionized the field of PSCs used as models of neurogenesis in two ways. Firstly, their somatic origin and the relative simplicity of the reprograming process has dramatically expanded the use of PSCs in general by removing the ethical constraint linked to embryo destruction. Secondly, iPSC derived from individuals with a known clinical profile represent real “patients in a dish” and offer for the first time the opportunity to address the role of miRNAs in the etiology of complex neuro-developmental disorders using patient-derived neurons. Many questions regarding the iPSC system remain to be answered and more particularly whether the reprograming step can actually compromise the proper modeling of a disease by erasing epigenetic signatures including the genuine miRNA expression profile.

While still in its infancy, the hPSC field has already demonstrated its usefulness to elucidate the function of miRNA in critical aspect of human neurogenesis. It should reveal its full potential in the coming years to become a standard that will complement studies performed in animal models.

## Conflict of Interest Statement

The authors declare that the research was conducted in the absence of any commercial or financial relationships that could be construed as a potential conflict of interest.
